# Proton‐Gated Ring‐Closure of a Negative Photochromic Azulene‐Based Diarylethene

**DOI:** 10.1002/anie.202007989

**Published:** 2020-08-19

**Authors:** Ian Cheng‐Yi Hou, Fabian Berger, Akimitsu Narita, Klaus Müllen, Stefan Hecht

**Affiliations:** ^1^ Synthetic Chemistry Max Planck Institute for Polymer Research Ackermannweg 10 55128 Mainz Germany; ^2^ Department Chemie Johannes Gutenberg-University Mainz Duesbergweg 10–14 55128 Mainz Germany; ^3^ Department of Chemistry & IRIS Adlershof Humboldt-Universität zu Berlin Brook-Taylor-Straße 2 12489 Berlin Germany; ^4^ Organic and Carbon Nanomaterials Unit Okinawa Institute of Science and Technology Graduate University 1919-1 Tancha, Onna-son Kunigami Okinawa 904-0495 Japan; ^5^ DWI—Leibniz Institute for Interactive Materials Forckenbeckstr. 50 52074 Aachen Germany; ^6^ Institute of Technical and Macromolecular Chemistry RWTH Aachen University Worringer Weg 2 52074 Aachen Germany

**Keywords:** acid-base equilibria, azulene, diarylethene, negative photochromism, photochemistry

## Abstract

Proton‐responsive photochromic molecules are attractive for their ability to react on non‐invasive rapid optical stimuli and the importance of protonation/deprotonation processes in various fields. Conventionally, their acidic/basic sites are on hetero‐atoms, which are orthogonal to the photo‐active π‐center. Here, we incorporate azulene, an acid‐sensitive pure hydrocarbon, into the skeleton of a diarylethene‐type photoswitch. The latter exhibits a novel proton‐gated negative photochromic ring‐closure and its optical response upon protonation in both open and closed forms is much more pronounced than those of diarylethene photoswitches with hetero‐atom based acidic/basic moieties. The unique behavior of the new photoswitch can be attributed to direct protonation on its π‐system, supported by ^1^H NMR and theoretical calculations. Our results demonstrate the great potential of integrating non‐alternant hydrocarbons into photochromic systems for the development of multi‐responsive molecular switches.

## Graphical abstract

Incorporation of an azulene moiety into a diarylethene photoswitch gives rise to unprecedented acid‐dependent negative photochromism. The unusual photochromic behavior can be understood by direct protonation on the photoactive π‐system due to the unique electronic structure of azulene.

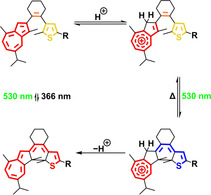

Stimuli‐responsive molecules are the basis of information processing in biological as well as synthetic complex systems. Among them, photochromic molecules are particularly attractive for the ability to convert between (at least) two states by noninvasive and rapid optical stimuli with a potential of high spatial and temporal resolution.^1^, ^2^, ^3^, ^4^, ^5^, ^6^ Additionally, multiple photochromic molecules have been developed,^7^, ^8^ which in addition to light respond to a secondary stimulus, such as the presence of ions,^9^, ^10^, ^11^, ^12^ oxidants/reductants,^13^, ^14^ or acids/bases.^15^, ^16^, ^17^ On the one hand, the second stimulus can gate the photochromism, leading to potential applications for example in logic devices. On the other hand, photochromism provides a means to remotely control specific molecular properties including polarity, oxidation/reduction potential as well as acidity/basicity. In particular, the response to changes in pH is appealing due to the generally rapid kinetics of protonation/deprotonation processes as well as the importance of proton gradients in biological signal transduction, energy conversion processes, supramolecular chemistry, and catalysis.

In principle, proton‐response can be implemented into a photochromic system by incorporation of an acidic/basic group in conjugation to^15^, ^18^, ^19^, ^20^, ^21^ or as part of^16^, ^17^, ^22^, ^23^, ^24^, ^25^ the photoactive reaction center. Commonly used moieties are heteroatom‐based, for example, pyridine^23^, ^26^, ^27^, ^28^, ^29^ or phenol.^16^, ^24^, ^30^, ^31^ The basic sites of these functional groups are located on the lone pairs of the heteroatoms, which are orthogonal to the π‐core (Figure 1 a), thereby limiting their influence on the photophysical behavior of the switch. In strong contrast, azulene, one of the most representative non‐alternant aromatic hydrocarbons, can be directly protonated on its π‐skeleton forming a vinyl substituted aromatic tropylium species^32^, ^33^ and thereby is converted from a 10π electron aromatic system into a (6+2)π electron one (Figure 1 b). Due to this drastic change on π‐conjugation upon protonation, incorporation of an azulene moiety into a photoswitchable molecule should lead to an unprecedented proton‐response since photochromism itself is also inherently based on altering π‐conjugation.

**Figure 1:**
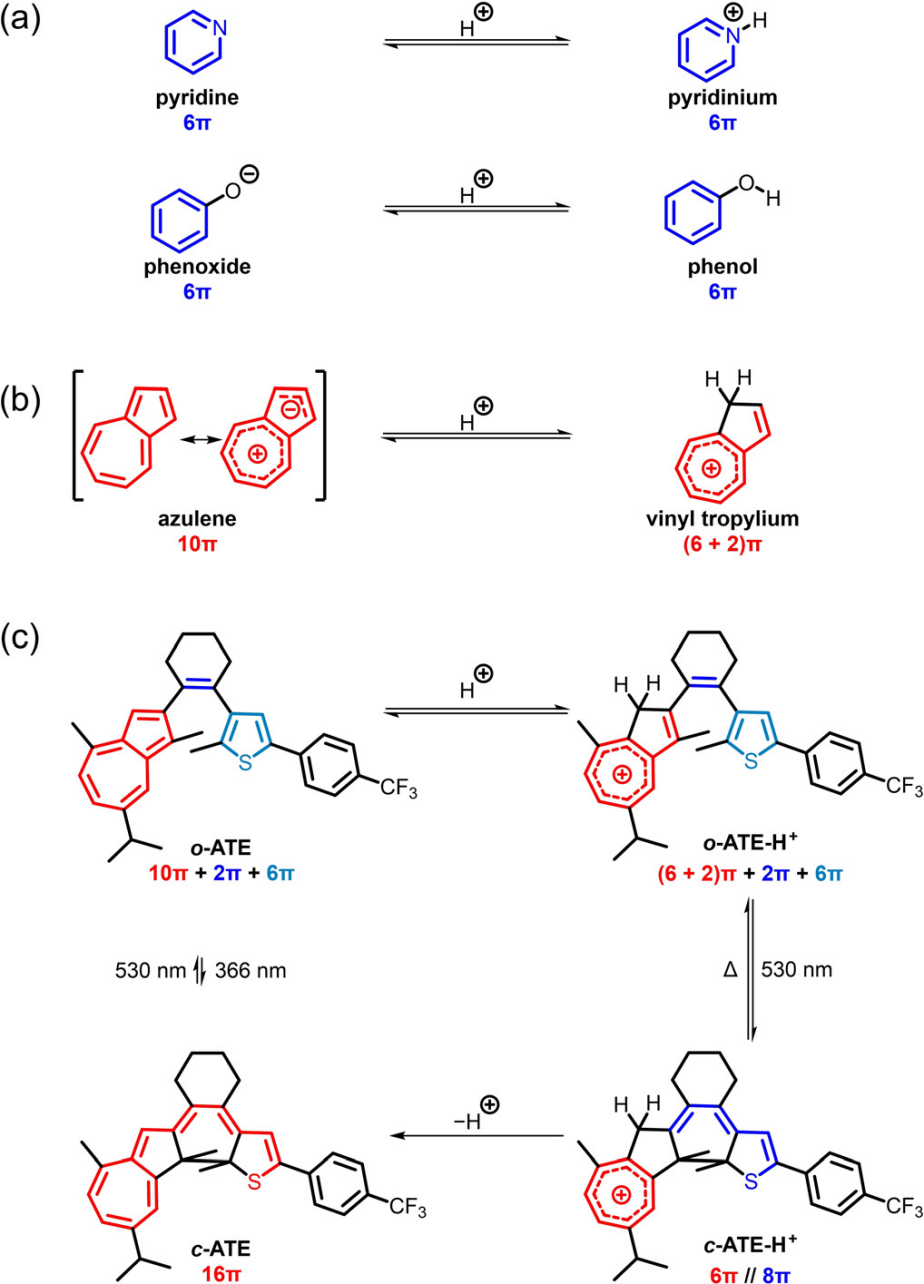
(a) Pyridine and phenoxide as well as their conjugated acids pyridinium and phenol, respectively. (b) Equilibrium between azulene (shown by two representative resonance structures) and the corresponding conjugated acid. (c) Proton‐gated photochromism of azulene‐based diarylethene **ATE** in its open (top) and closed (bottom) isomers and their conjugated acids.

Thus far, azulene has rarely been employed in the skeleton of photoswitches,^34^, ^35^, ^36^, ^37^, ^38^ and their proton‐responsive photochromism was only sporadically studied in cyanostilbene‐type photoswitches.^39^, ^40^, ^41^ To unravel the full potential of the proton‐response of azulene in the development of novel multi‐stimuli responsive molecular photoswitches, here we describe the effect of incorporating azulene into the skeleton of a diarylethene‐type (DAE) photoswitch on its photochromic behavior. In this work, we synthesized azulenylthienylethene (**ATE**) where one of the aryl groups originates from guaiazulene, a naturally occurring and commercially available terpenoid azulene derivative (Figure 1 c). In strong contrast to most DAE‐type photoswitches, which exhibit positive photochromism because of an extended conjugated system in the closed form, photocyclization of the protonated open isomer, ***o*** **‐ATE‐H^+^**, leads to a 100 nm hypsochromic shift of the strongest visible light absorption band, and thus constitutes an example of negative photochromism. Moreover, protonation of ***o*** **‐ATE** and deprotonation of ***c*** **‐ATE‐H^+^** causes more than 120 nm hypsochromic shift for their S_0_→S_1_ transitions, which is much more significant than DAE‐type photoswitches employing heteroatom‐based acid/base functional groups upon protonation/deprotonation. Furthermore, **ATE** exhibits pronounced proton‐gated photochromism in which the protonated open isomer ***o*** **‐ATE‐H^+^** undergoes more efficient photocyclization as compared to its conjugated base ***o*** **‐ATE**. Our results demonstrate the great potential incorporating azulene in photochromic molecules for the development of multiple‐stimuli responsive molecular photoswitches.

Synthesis of ***o*** **‐ATE** was accomplished by an initial Pd‐catalyzed cross‐coupling^42^ between cyclohexanone and 3‐bromo‐2‐methyl‐5‐[4‐(trifluoromethyl)phenyl]thiophene^43^ followed by conversion into the corresponding enol triflate. Subsequently, the guaiazulenyl moiety was introduced via Suzuki coupling involving 2‐guaiazulenylboronic acid pinacol ester^44^ (see Scheme S1). The desired ***o*** **‐ATE** exhibits a weak (*ϵ*≈200 m ^−1^ cm^−1^) and broad (480–750 nm) visible light absorption band (Figure 2 a, black line), which corresponds to a typical S_0_→S_1_ transition of azulene derivatives.^45^, ^46^ Furthermore, the “blue window” of azulene appears at 430–480 nm.

**Figure 2:**
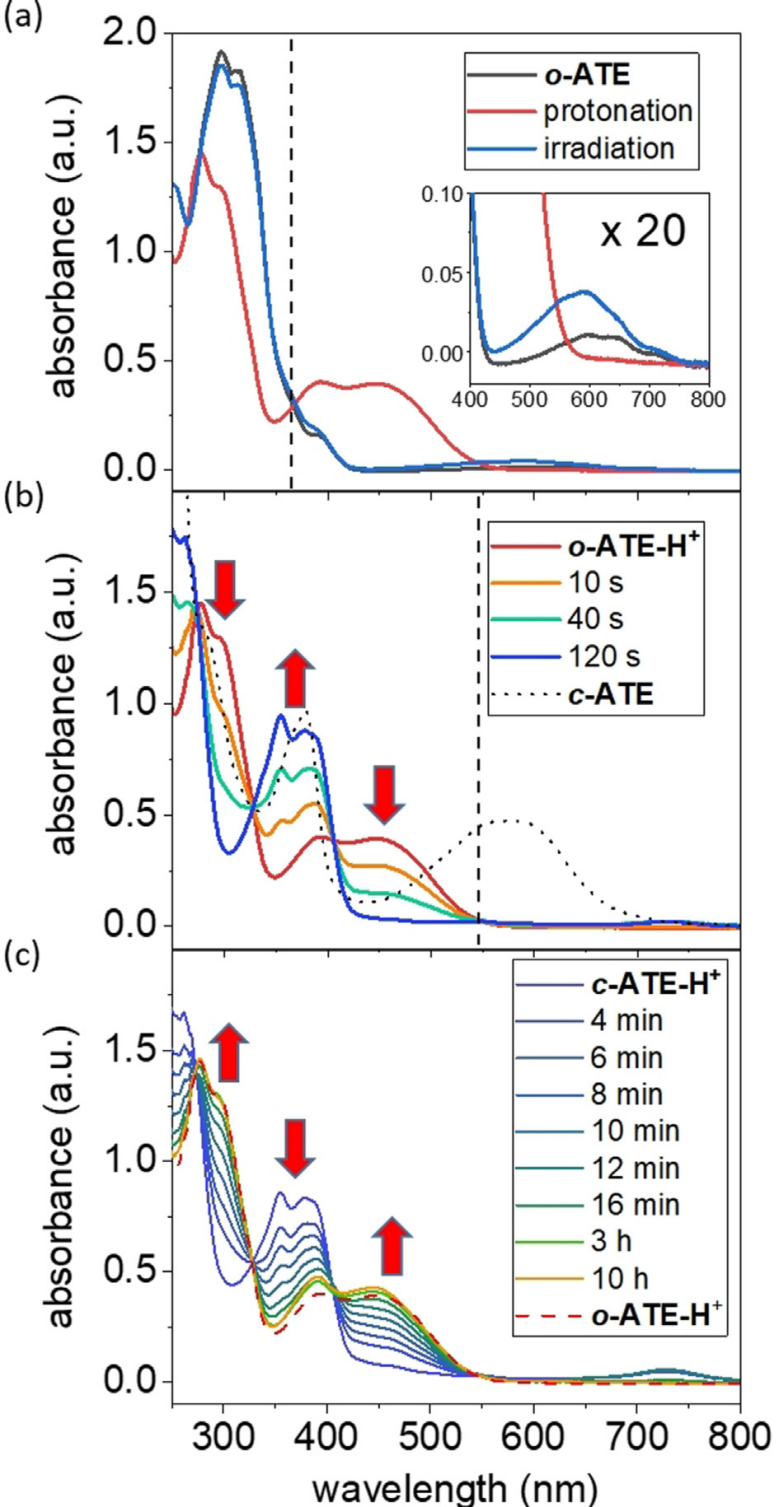
UV/Vis absorption spectra of (a) ***o*** **‐ATE** (5.0×10^−5^ m in cyclohexane, black) either after irradiation at 365 nm for 5 min (blue) or after addition of TFA (3.0×10^−2^ m) to form ***o*** **‐ATE‐H^+^** (red). (b) ***o*** **‐ATE‐H^+^** (red) upon irradiation at 546 nm for 2 min at −30 °C to yield ***c*** **‐ATE‐H^+^** (blue) and subsequent addition of excess triethylamine (9.0×10^−2^ m) to form ***c*** **‐ATE** (black). (c) Thermal ring‐opening of ***c*** **‐ATE‐H^+^** to ***o*** **‐ATE‐H^+^** at room temperature. Irradiation wavelengths are indicated by dash lines.

Using these characteristic absorption features, the response of ***o*** **‐ATE** to various irradiation conditions and the addition of acid was examined. Photochromism of ***o*** **‐ATE** appears rather inefficient since irradiation at 365 nm for 5 min leads to only minor spectral changes (Figure 2 a, blue line). Notably, the absorption band centered at around 600 nm (Figure 2 a, inset) increases and is accompanied by a decrease of the UV absorption. This constitutes a typical spectral feature of DAE‐type photoswitches during photocyclization,^7^, ^8^ suggesting the formation of ***c*** **‐ATE**. Upon prolonged irradiation, the spectra continue to follow this trend with a clean isosbestic point at 343 nm, however, even after irradiating for 70 min the photostationary state (PSS) is not reached (see Figure S1 b). Alternative irradiation of the S_0_→S_1_ transition wavelength of azulene using a 660 nm LED only led to negligible spectral changes despite its long duration of 16 h (see Figure S1 a). Attempts to switch back the in situ formed ring‐closed ***c*** **‐ATE** by irradiation at 546 nm were not successful as well (see Figure S1 c). These results suggest very low quantum yields for both, ring‐closure as well as ring‐opening of ***o*** **‐ATE** and ***c*** **‐ATE**, respectively.

When ***o*** **‐ATE** is exposed to trifluoroacetic acid (TFA), its UV absorption at around 300 nm weakens and blue‐shifts while a strong absorption band extending from 350 to 550 nm appears (Figure 2 a, red line). The observed spectral behavior resembles that of guaiazulene derivatives upon protonation.^47^ The occurrence and site of protonation were verified by ^1^H NMR spectroscopy (Figure 3 a,b).^47^, ^48^, ^49^ Importantly, the characteristic proton signal of the 1‐position of ***o*** **‐ATE** (indicated by the red number in Figure 3 a), initially located in the aromatic region at 7.0 ppm (Figure 3 a), disappears in ***o*** **‐ATE‐H^+^** upon protonation (Figure 3 b). Instead, an allylic proton signal with doubled relative intensity appears at 3.9 ppm, suggesting exclusive protonation of the 1‐position. Such ^1^H NMR spectral change typically indicates protonation of azulene derivatives to form tropylium species.^47^, ^49^ It is worth noting that the proton signals of ***o*** **‐ATE** completely vanish in the ^1^H NMR spectrum of ***o*** **‐ATE‐H^+^** (Figure 3 b) while the S_0_→S_1_ transition of azulene disappears in the UV/Vis absorption spectrum of ***o*** **‐ATE‐H^+^** (Figure 2 a, inset). These spectral changes indicate a complete conversion of ***o*** **‐ATE** to ***o*** **‐ATE‐H^+^** upon TFA addition. Over a period of 16 h in the dark no significant UV/Vis absorption spectral changes of ***o*** **‐ATE‐H^+^** were observed (see Figure S2), showing reasonable thermal stability of ***o*** **‐ATE‐H^+^** at room temperature. However, over longer timeframes (one week) decomposition was observed.

**Figure 3:**
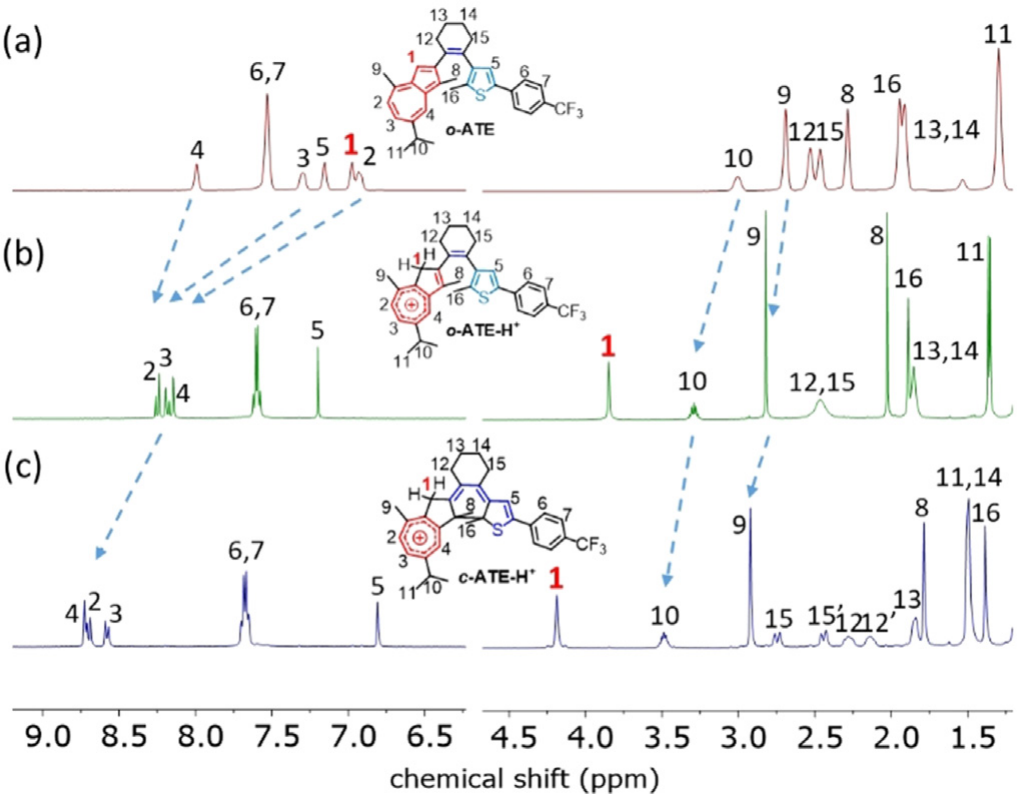
^1^H NMR spectra of CD_2_Cl_2_ solution of: (a) ***o*** **‐ATE** at 298 K, (b) ***o*** **‐ATE‐H^+^** at 248 K in the presence of 16 equiv TFA, and (c) ***c*** **‐ATE‐H^+^** at 248 K generated by in situ irradiating (b) at 565 nm.

Remarkably and in strong contrast to ***o*** **‐ATE**, photochromism of its protonated species ***o*** **‐ATE‐H^+^** is significantly altered and its efficiency is largely improved. When a solution of ***o*** **‐ATE‐H^+^** is exposed to 546 nm irradiation, its UV/Vis absorption spectrum completely changes within 2 min (Figure 2 b). Moreover, the strongest visible light absorption band (absorption edge at around 415 nm) of the formed photoreaction product exhibits a marked blue‐shift by more than 100 nm from that of ***o*** **‐ATE‐H^+^** (absorption edge at around 545 nm) (Figure 2 b). Therefore, ***o*** **‐ATE‐H^+^** almost fully decolorizes after the photoreaction and exhibits negative photochromism. Importantly, clear isosbestic points are observed, indicating a clean two‐state interconversion, which is further supported by the corresponding extinction difference diagrams (see Figure S3). The nature of the photochemical product is revealed once again by ^1^H NMR spectroscopy. Upon irradiating a CD_2_Cl_2_ solution of ***o*** **‐ATE‐H^+^** at 565 nm, a gradual ^1^H NMR spectral change is observed with a clean two‐species transition (see Figure S4), leading to quantitative formation of ***c*** **‐ATE‐H^+^** by photochemical ring‐closure of ***o*** **‐ATE‐H^+^** (Figure 3 c). Interestingly, after formation of ***c*** **‐ATE‐H^+^** no proton transfer takes place from an allylic proton in the 1‐position to other positions to re‐establish π‐conjugation between the tropylium residue and the cyclohexadiene core, although quantum chemical calculations predict that the most basic site of ***c*** **‐ATE** is not on the skeleton of guaiazulene but on the thiophene fragment with a possible −48 kJ mol^−1^ energy gain upon proton transfer (see Figure S5). Instead, the tropylium cation in ***c*** **‐ATE‐H^+^** is isolated and hence its positive charge is more localized in comparison with that of ***o*** **‐ATE‐H^+^**. As a result, the signals of protons on the guaiazulene skeleton are further downfield‐shifted in the ^1^H NMR spectrum of ***c*** **‐ATE‐H^+^** as compared to that of ***o*** **‐ATE‐H^+^** (see blue arrows in Figures 3 b,c). Notably, despite isolation of the tropylium moiety from the rest of the conjugated π‐system, a slight interaction between the tropylium and the cyclohexene remains, which is indicated by the weak S_0_→S_1_ transition of ***c*** **‐ATE‐H^+^** centered at 730 nm in its UV/Vis absorption spectrum (Figure 2 b). This transition has a charge transfer character that donates electron density from the thiophene‐cyclohexene skeleton to the tropylium residue, as supported by quantum chemical calculations (see Figure S6).

The closed isomer ***c*** **‐ATE‐H^+^** undergoes thermal ring‐opening reaction with a half‐life of around 7 min at room temperature (Figure 2 c), similar to reported proton‐responsive DAE‐type photoswitches.^21^, ^22^, ^42^ Notably, the reaction did not lead to the exact same absorption spectrum of ***o*** **‐ATE‐H^+^**, suggesting the thermal back reaction was accompanied with some decomposition of **ATE** (Figure 2 c and S2). Photochemical ring‐opening of ***c*** **‐ATE‐H^+^** by irradiating at 365 nm or 740 nm was found to be inefficient (see Figure S7). Importantly, in situ deprotonation of ***c*** **‐ATE‐H^+^** with triethylamine provided the neutral closed isomer ***c*** **‐ATE**, which exhibits a typical absorption band at 450–700 nm of the closed form of DAE‐type photoswitches (Figure 2 b, black dotted line).^7^, ^8^ This absorption band is slightly more red‐shifted than the one of ***c*** **‐ATE** obtained by direct photoreaction of ***o*** **‐ATE** (see Figure S8). The deviation may be explained by considering the fact that direct photochemical ring‐closure of ***o*** **‐ATE** could lead to a mixture of two regioisomers, whereas photoreaction of ***o*** **‐ATE‐H^+^** is regioselective (see Figure S9). There are only negligible UV/Vis absorption spectral changes for a solution of ***c*** **‐ATE** kept in the dark at room temperature for 7 h (see Figure S10). Thus, and unlike ***c*** **‐ATE‐H^+^**, the neutral form ***c*** **‐ATE** is thermally stable. However, photochemical ring‐opening of ***c*** **‐ATE** is still inefficient (see Figure S11). The assigned molecular structures of both pairs of switching states, that is, ***o*** **‐ATE**, ***o*** **‐ATE‐H^+^**, ***c*** **‐ATE**, and ***c*** **‐ATE‐H^+^**, are further supported by calculated UV/Vis absorption spectra (see Figure S12), which are in reasonable agreement with experimental spectra.

Although negative photochromic^50^, ^51^ and proton‐gated photochemical^18^, ^21^, ^23^, ^24^, ^28^, ^52^ ring‐closure have been separately reported for different DAE‐type photoswitches, to the best of our knowledge, **ATE** is the first example that undergoes a proton‐gated negative photochromic ring‐closure reaction. In addition, protonation of ***o*** **‐ATE** and deprotonation of ***c*** **‐ATE‐H^+^** causes pronounced spectral changes, where the absorption edges of S_0_→S_1_ transitions exhibit hypsochromic shifts in both cases by more than 120 nm (Figures 2 a,b). These spectral shifts are considerably larger than those of the other proton‐responsive DAE‐type photoswitches using heteroatom‐based acid/base functional groups, which typically exhibit spectral shifts smaller than 50 nm upon protonation/deprotonation.^34^, ^35^, ^36^, ^37^, ^38^, ^39^, ^40^, ^41^ Although proton‐response of azulene incorporated photoswitches has been sporadically reported,^39^, ^40^, ^41^ our ^1^H NMR analyses convincingly suggest that the unique features of our system stem from protonation of the azulene moiety, which is *directly* incorporated in the photoreactive core. Most importantly, π‐conjugation between the tropylium moiety and the residual π‐system is strongly diminished after transformation of ***o*** **‐ATE‐H^+^** into ***c*** **‐ATE‐H^+^** (Figure 3 c), giving rise to the negative photochromic ring‐closure that has not yet been reported for a regular DAE‐type photoswitch.

The unique photochemical behavior of **ATE** and its strong dependence on protonation can be explained by the nature of the S_0_→S_1_ transitions of ***o*** **‐ATE** and ***o*** **‐ATE‐H^+^** (see Figure S13 and Table S1). The S_0_→S_1_ transition of the protonated form ***o*** **‐ATE‐H^+^** is 35 times more intense than that of ***o*** **‐ATE**. More importantly, the S_0_→S_1_ transition of ***o*** **‐ATE** is a local charge transfer that shifts electron density from the five‐membered ring onto the seven‐membered ring of azulene (Figure 4). This transition casts minor influence on the electron density in the triene photoreaction center of DAE‐type photoswitch. Similarly, the S_0_→S_1_ transition of ***o*** **‐ATE‐H^+^** shifts electron density to the seven‐membered ring of azulene. However, the electron density on the five‐membered ring of azulene has been already largely reduced by protonation. As a result, the S_0_→S_1_ transition of ***o*** **‐ATE‐H^+^** leads to further depopulation of orbitals that resemble the double bonds of the triene center (Figure 4). This depopulation resembles the electron density reorganization that occurs during ring‐closure of DAE‐type photoswitches and effectively prepares a similar electron density distribution as present in the closed isomer. A subsequent relaxation of the electron density that recovers the aromaticity of the tropylium moiety can then lead to the formation of a carbon‐carbon bond and explain the much higher photoreactivity of ***o*** **‐ATE‐H^+^** in comparison with that of ***o*** **‐ATE**.

**Figure 4:**
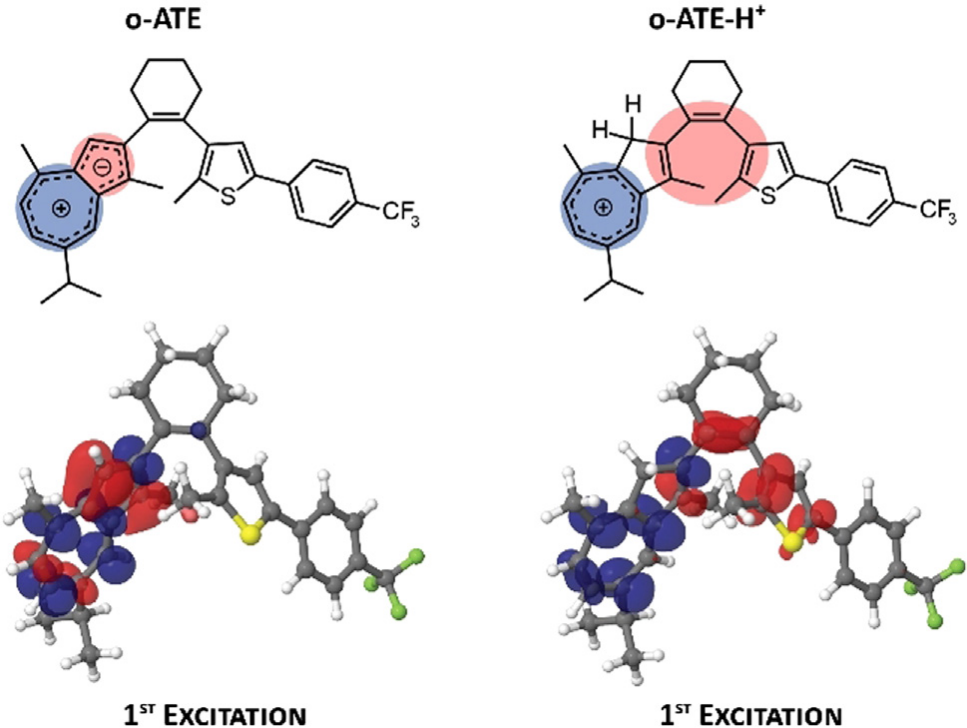
Schematic and calculated electron density changes (electron difference densities) of the S_0_→S_1_ transition of ***o*** **‐ATE** and ***o*** **‐ATE‐H^+^**. Red/blue lobes refer to decrease/increase of electron density during excitation, respectively.

In conclusion, we synthesized a novel DAE‐type photoswitch by incorporating an acid‐sensitive azulene moiety directly into the photoreactive skeleton. While photochemical ring closure and opening in the charge‐neutral state are rather inefficient, protonation of the azulene moiety largely improves photoefficiency and induces drastic changes in the optical spectra, thereby giving rise to a negative photochromism. This proton‐gated negative photochromic behavior should prove particularly useful for applications in optical memories, where data can be written and erased using a photoactive, thermally labile state (the protonated form) and read nondestructively in a photoinactive state (the neutral form). The results highlight the beneficial effect of integrating the unique azulene moiety on the photochromic behavior of DAE‐type photoswitches and open opportunities for designing new multi‐stimuli responsive photochromic materials.

## Conflict of interest

The authors declare no conflict of interest.

## Supporting information

As a service to our authors and readers, this journal provides supporting information supplied by the authors. Such materials are peer reviewed and may be re‐organized for online delivery, but are not copy‐edited or typeset. Technical support issues arising from supporting information (other than missing files) should be addressed to the authors.

Supplementary
