# Supplementary material for: Proton‐Gated Ring‐Closure of a Negative Photochromic Azulene‐Based Diarylethene
Source: Angew Chem Int Ed Engl. 2020 Aug 19;59(42):18532–6. doi: 10.1002/anie.202007989 (PMC7589205; doi:10.1002/anie.202007989)
Supplement: Supplementary file 1 — Supplementary [file ANIE-59-18532-s001.pdf]

Angewandte Chemie
Eine Zeitschrift der Gesellschaft Deutscher Chemiker

Supporting Information

Proton-Gated Ring-Closure of a Negative Photochromic Azulene-Based Diarylethene

Ian Cheng-Yi Hou, Fabian Berger, Akimitsu Narita, Klaus Müllen,* and Stefan Hecht**

anie_202007989_sm_miscellaneous_information.pdf

Table of Contents

**General Information**	S2
**Synthesis of ATE**	S2
**Photochemical Methods**	S5
**Computational Details**	S5
**Complementary Figures**	S7
**NMR Spectra of New Compounds**	S16
**References**	S20

General Information

Unless otherwise noted, materials were purchased from Fluka, Aldrich, Acros, abcr, Merck, and other commercial suppliers, and were used as received unless otherwise specified. 3-Bromo-2-methyl-5-[4-(trifluoromethyl)phenyl]thiophene was synthesized following ref[1]. 2-Guaiazulenylboronic acid pinacol ester was synthesized following ref[2]. All reactions working with air- or moisture-sensitive compounds were carried out under argon atmosphere using standard Schlenk line techniques. Preparative column chromatography was performed on silica gel from Merck with a grain size of 0.04–0.063 mm (flash silica gel, Geduran Si 60). Melting points were determined on a Büchi hot stage apparatus without correction. NMR spectra were recorded in deuterated solvents using Bruker AVANCE III 300 and AVANCE III 500 spectrometers. Chemical shifts (*δ*) were expressed in ppm relative to the residual of solvent (CD_2Cl_2 @ 5.32 ppm for ^{1}H NMR, 53.84 ppm for ^{13}C NMR). Coupling constants (*J*) were recorded in Hertz (Hz) with multiplicities explained by the following abbreviations: s = singlet, d = doublet, t = triplet, q = quartet, dd = doublet of doublets, dt = doublet of triplets, m = multiplet, br = broad. The spin-echo attached-proton test (APT) ^{13}C NMR spectrum was recorded with C, CH_2 showing negative signal and CH, CH_3 showing positive signal. The UV–Vis absorption spectra were measured with a Perkin-Elmer Lambda 900 spectrophotometer in a quartz cuvette (Hellma) with a light path of 1 cm at room temperature. High-resolution mass spectra (HRMS) were recorded by matrix-assisted laser desorption/ionization (MALDI) using *trans*-2-[3-(4-tert-Butylphenyl)-2-methyl-2-propenylidene]malononitrile (DCTB) as matrix with a Bruker Reflex II-TOF spectrometer (MALDI-TOF).

Synthesis of ATE

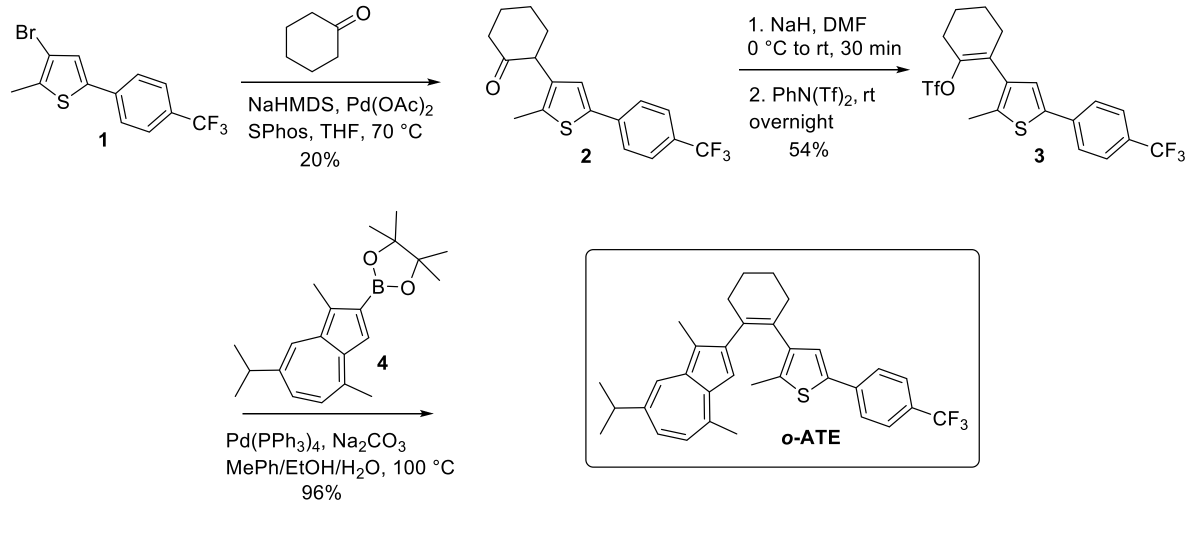

Scheme S1. Synthesis of ***o*-ATE**. Reagents: NaHMDS = sodium hexamethyldisilazide; SPhos = 2-dicyclohexylphosphino-2',6'-dimethoxybiphenyl; THF = tetrahydrofuran; DMF = dimethylformamide; $PhN(Tf)_2$ = *N*-Phenyl-bis(trifluoromethanesulfonimide).

Synthesis of the target compound ***o*-ATE** was performed through stepwise Pd-catalyzed coupling reactions (Scheme S1). First, adapting a literature reported coupling reaction involving cyclohexenolate,[3] the cyclohexene bridge was connected with thienyl building block **1**[1] to form ketone **2**, followed by a functional group transformation that leads to **3** equipped with a reactive triflate functional group. The coupling step was not efficient with only 20% yield of **2**, accompanied by isolation of 56% debrominated **1**, agreed with previous observation.[3] Then, the azulenyl moiety was introduced by a Suzuki coupling of **3** with 2-guaiazulenylboronic acid pinacol ester **4**, which can be synthesized from selective borylation of pristine guaiazulene,[2] to afford ***o*-ATE** in an outstanding 96% yield. The structure of ***o*-ATE** was unambiguously characterized by its ^{1}H NMR, ^{13}C NMR, and HR-MALDI-TOF spectra.

*Synthesis of 2-{2-methyl-5-[4-(trifluoromethyl)phenyl]thiophen-3-yl}cyclohexan-1-one (**2**)*: Synthesis of **2** was accomplished applying an adapted literature-reported procedure for a similar structure.[3] A mixture of 3-bromo-2-methyl-5-[4-(trifluoromethyl)phenyl]thiophene[1] (**1**) (1.00 g, 3.11 mmol), cyclohexanone (1.35 mL, 13.1 mmol), Pd(OAc)$_2$ (36 mg, 0.16 mmol), SPhos (131 mg, 0.319 mmol), and tetrahydrofuran (35 mL) was degassed by freeze-pump-thaw technique for two cycles. Sodium bis(trimethylsilyl)amide (13.1 mL, 1 M in tetrahydrofuran , 13.1 mmol) was then injected, and the mixture was heated at 70 °C under vigorously stirring overnight. The mixture was then allowed to cool down to room temperature, and diluted with ether and water. The organic phases was washed with water, brine, and dried over MgSO$_4$. The solvent was then removed *in vacuo* and the residue was purified by silica gel column chromatography (gradient, dichloromethane/petroleum ether = 1/3 to 1/1). 2-Methyl-5-(4-(trifluoromethyl)phenyl)thiophene was isolated as the first band (420 mg, 56%) and the target product **2** as the second band (white solid, 243 mg, 20%): mp 107.5–112.7 °C; ^{1}H NMR (300 MHz, CD$_2$Cl$_2$, *δ*): 7.67 (d, *J* = 8.4 Hz, 2H), 7.60 (d, *J* = 8.4 Hz, 2H), 7.19 (s, 1H), 3.66 (dd, *J* = 12.3, 5.4 Hz, 1H), 2.59–2.44 (m, 2H), 2.33 (s, 3H), 2.29–2.12 (m, 2H), 2.09–1.93 (m, 2H), 1.93–1.74 (m, 2H); ^{13}C NMR (75 MHz, CD$_2$Cl$_2$, δ) 209.38, 138.48, 137.78, 137.26, 136.59, 128.66 (q, *J* = 33 Hz), 126.12 (q, *J* = 4 Hz), 125.96, 125.66, 124.78 (q, *J* = 272 Hz), 51.10, 42.69, 35.50, 28.09, 26.02, 13.48; HRMS (MALDI-TOF) *m/z*: [M]$^+$ calcd for $C_{26}H_{17}Br$ 338.0952; Found [M]$^+$ 338.0888.

*Synthesis of 3-[2-(7-isopropyl-1,4-dimethylazulen-2-yl)cyclohex-1-en-1-yl]-2-methyl-5-[4-(trifluoromethyl)phenyl]thiophene (**o-ATE**)*: To a suspension of NaH (39 mg, 60% w/w, ~ 1 mmol) on dimethylformamide (3 mL) was injected a dimethylformamide (6 mL) solution of 2-{2-methyl-5-[4-(trifluoromethyl)phenyl]thiophen-3-yl}cyclohexan-1-one (**2**) (174 mg, 0.514 mmol) at 0 °C. The mixture was gradually warmed up to room temperature and stirred for 30 min. *N*-phenyl-trifluoromethanesulfonimide (222 mg, 0.622 mg) was then added in one portion, and the mixture was allowed to react overnight. Diethyl ether and water was then added into the mixture. The organic layer was washed with water, brine, and dried over MgSO$_4$. After solvent was removed *in vacuo*, the residue was purified by silica gel column chromatography (dichloromethane/petroleum ether = 1/6) to afford 2-{2-methyl-5-[4-(trifluoromethyl)phenyl]thiophen-3-yl}cyclohex-1-en-1-yl trifluoromethanesulfonate (**3**) as a

white solid (131 mg, 54%, ^{1}H NMR (300 MHz, CD_2Cl_2, δ): 7.66 (d, *J* = 8.5 Hz, 2H), 7.61 (d, *J* = 8.5 Hz, 2H), 7.15 (s, 1H), 2.55–2.44 (m, 2H), 2.44–2.39 (m, 2H), 2.37 (s, 3H), 1.88 (q, *J* = 7.0, 6.2 Hz, 2H), 1.78 (q, *J* = 7.0 Hz, 2H).), which was directly used in the next step without further purification. A mixture of **3** (90 mg, 0.19 mmol), 2-guaiazulenylboronic acid pinacol ester[2] (250 mg, 0.77 mmol), Na_2CO_3 (82 mg, 0.77 mmol), water (1.5 mL), EtOH (1.5 mL), and toluene (3 mL) was degassed by freeze-pump-thaw technique for one cycle. $Pd(PPh_3)_4$ (11 mg, 9.6 µmol) was then added, and the mixture was further degassed by freeze-pump-thaw technique for another two cycles. The mixture was then heated at 100 °C overnight under vigorous stirring. The mixture was then allowed to cool down to room temperature, and diluted with diethyl ether and water. The organic phase was washed with water, brine, and dried over $MgSO_4$. Solvent was then removed *in vacuo* and the residue was purified by silica gel column chromatography (dichloromethane/petroleum ether = 1/20) to afford ***o*-ATE** as a blue solid (95 mg, 52% over two steps): mp 115.7–117.9 °C; ^{1}H NMR (500 MHz, CD_2Cl_2, δ): 8.04–7.95 (br, 1H), 7.62–7.47 (br, 4H), 7.35–7.24 (br, 1H), 7.20–7.11 (br, 1H), 7.03–6.95 (br, 1H), 6.95–6.87 (br, 1H), 3.08–2.93 (br, 1H), 2.77–2.63 (br, 3H), 2.60–2.50 (br, 2H), 2.50–2.40 (br, 2H), 2.34–2.23 (br, 3H), 2.02–1.93 (br, 3H), 1.93–1.84 (br, 4H), 1.40–1.22 (br, 6H); ^{13}C NMR (126 MHz, CD_2Cl_2, δ): 151.99, 142.87, 142.83, 140.52, 138.59, 137.34, 137.11, 136.38, 136.06, 134.90, 133.78, 132.67, 131.51, 128.43 (q, *J* = 32 Hz), 127.08, 126.05 (q, *J* = 4 Hz), 125.46, 125.21, 124.79 (q, *J* = 272 Hz), 122.38, 114.05, 38.67, 32.62, 32.17, 30.11, 24.86, 24.11, 23.68, 23.66, 14.45, 11.67; HRMS (MALDI-TOF) *m/z*: $[M]^+$ calcd for $C_{26}H_{17}Br$ 518.2255; Found $[M]^+$ 518.2261.

***o*-ATE-H$^+$**: ^{1}H NMR (500 MHz, CD_2Cl_2 + 16 eq. trifluoroacetic acid, δ): 8.25 (d, *J* = 11.2 Hz, 1H), 8.18 (dd, *J* = 11.2, 1.9 Hz, 1H), 8.15 (d, *J* = 1.9 Hz, 1H), 7.61 (d, *J* = 8.6 Hz, 2H), 7.59 (d, *J* = 8.6 Hz, 2H), 7.20 (s, 1H), 3.85 (s, 2H), 3.29 (sept, *J* = 6.8 Hz, 1H), 2.82 (s, 3H), 2.60–2.33 (br, 4H), 2.03 (s, 3H), 1.89 (s, 3H), 1.88–1.79 (br, 4H), 1.36 (d, *J* = 6.8 Hz, 6H).

***c*-ATE-H$^+$**: ^{1}H NMR (500 MHz, CD_2Cl_2 + 16 eq. trifluoroacetic acid, δ): 8.73 (s, 1H), 8.70 (d, *J* = 10.8 Hz, 1H), 8.58 (d, *J* = 10.8 Hz, 1H), 7.69 (d, *J* = 8.7 Hz, 2H), 7.66 (d, *J* = 8.7 Hz, 2H), 6.81 (s, 1H), 4.19 (s, 2H), 3.49 (sept, *J* = 6.7 Hz, 1H), 2.92 (s, 3H), 2.75 (d, *J* = 16.5 Hz, 1H), 2.44 (d, *J* = 15.6 Hz, 1H), 2.34–2.22 (br, 1H), 2.19–2.08 (br, 1H), 1.90–1.81 (br, 2H), 1.79 (s, 3H), 1.53–1.45 (m, 8H), 1.39 (s, 3H).

Photochemical Methods

The photochemical reaction in solution was conducted by direct irradiation of a cyclohexane solution of **ATE** in a quartz cuvette used for UV-vis absorption spectral measurement. The absorption spectra were directly recorded using a UV-Vis spectrophotometer after irradiation. The light source used was a Mercury arc lamp (HBO 200W/2, OSRAM). The irradiation wavelength was controlled by using optical filters (Schott Glaswerke). The 365 nm light irradiation was achieved by using a 3 mm GG320 combined with a UG11 filter to eliminate the high energy UV peaks and the visible light peaks of the light source to reveal only the 365 nm peak (Pd = 70 mW/cm^2). The 546 nm light irradiation was achieved by using 3 mm GG420 filter to block the UV light from the light source (Pd = 205 mW/cm^2). Lower energy peaks of the light source were not blocked. The light source used for ***o*-ATE-H$^+$** in CD_2Cl_2 for tracking the photochemical ring-closure reaction by ^{1}H NMR spectra was a 565 nm LED (Thorlab, Pd = 39 mW/cm^2). The light source was connected to the NMR tube *via* an optical fiber and the solution was irradiated *in-situ*. Because the solution used for NMR was 100-times more concentrated than that for UV-Vis experiments, and the power of the light source was lower, the irradiation time was much longer.

Computational Details

Structure optimizations were performed with ORCA 4.2.1,[4,5] employing the DFT functional B3-LYP[6,7] and empirical dispersion correction (D3),[8] a def2-TZVPP basis set,[9] the RI approximation, C1 symmetry, a SCF convergence criterion of 10^{-8} E_H and an optimization threshold of 10^{-7} E_H A^{-1}. Solvent effects were included with the conductor-like polarizable continuum model using the COSMO like epsilon function (cyclohexane: dielectric constant=2.02, refractive index = 1.43). Convergence of minimum structures of **o-ATE**, **o-ATE-H$^+$**, **c-ATE**, **c-ATE$_{C1}$-H$^+$** and **c-ATE$_{C16}$-H$^+$** has been verified by harmonic vibrational analysis, yielding no imaginary frequencies.

UV-vis excitation spectra and electron difference densities were calculated with Turbomole 7.0[10] in C1 symmetry, using the ADC(2) method, a def2-SVP basis set[11] and the implicit solvent model COSMO (cyclohexane: dielectric constant=2.02, refractive index = 1.43) without any reoptimization. A SCF threshold of 10^{-8} E_H has been used to calculate the lowest twelve excitations. Despite the small basis set, def2-SVP, these settings have been shown to provide sufficiently accurate excitation energies and UV-vis spectra.[12] Figures depicting structures based on B3-LYP+D3 or electron difference densities based on ADC(2) calculations were obtained *via* Jmol.[13] All structures are available as xyz-files in the compressed folder structures.zip.

Table S1. Excitation energies in nm and normalized oscillator strengths, f, obtained *via* ADC(2)//def2-SVP single point calculations based on B3-LYP+D3//def2-TZVPP optimized structures including cyclohexane as implicit solvent model.

Excitation		o-ATE	o-ATE-H^+	c-ATE	c-ATE_{C1}-H^+	c-ATE_{C16}-H^+
**1**	**Energy**	588	508	620	756	493
	**Intensity**	0.00578	0.20305	0.18842	0.00242	0.41159
**2**	**Energy**	355	422	529	682	428
	**Intensity**	0.07816	0.00653	0.05426	0.00192	0.00710
**3**	**Energy**	311	382	390	401	388
	**Intensity**	0.36835	0.08287	0.00376	0.54808	0.06712
**4**	**Energy**	294	327	364	359	333
	**Intensity**	0.11358	0.00203	0.34371	0.00411	0.00235
**5**	**Energy**	286	320	311	342	320
	**Intensity**	0.02636	0.07264	0.01998	0.00347	0.13701
**6**	**Energy**	275	287	300	293	282
	**Intensity**	0.07769	0.00326	0.02109	0.04098	0.01665
**7**	**Energy**	270	283	288	283	277
	**Intensity**	0.07691	0.00093	0.03330	0.10063	0.01714
**8**	**Energy**	256	267	274	281	270
	**Intensity**	0.00340	0.32436	0.04930	0.00245	0.00426
**9**	**Energy**	254	264	266	269	266
	**Intensity**	0.03432	0.15780	0.00627	0.06108	0.00232
**10**	**Energy**	250	253	258	268	261
	**Intensity**	0.16799	0.06558	0.10538	0.03394	0.16487
**11**	**Energy**	244	252	252	264	260
	**Intensity**	0.00854	0.04982	0.15840	0.05929	0.09496
**12**	**Energy**	231	251	244	260	255
	**Intensity**	0.03892	0.03114	0.01613	0.14163	0.07461

Complementary Figures

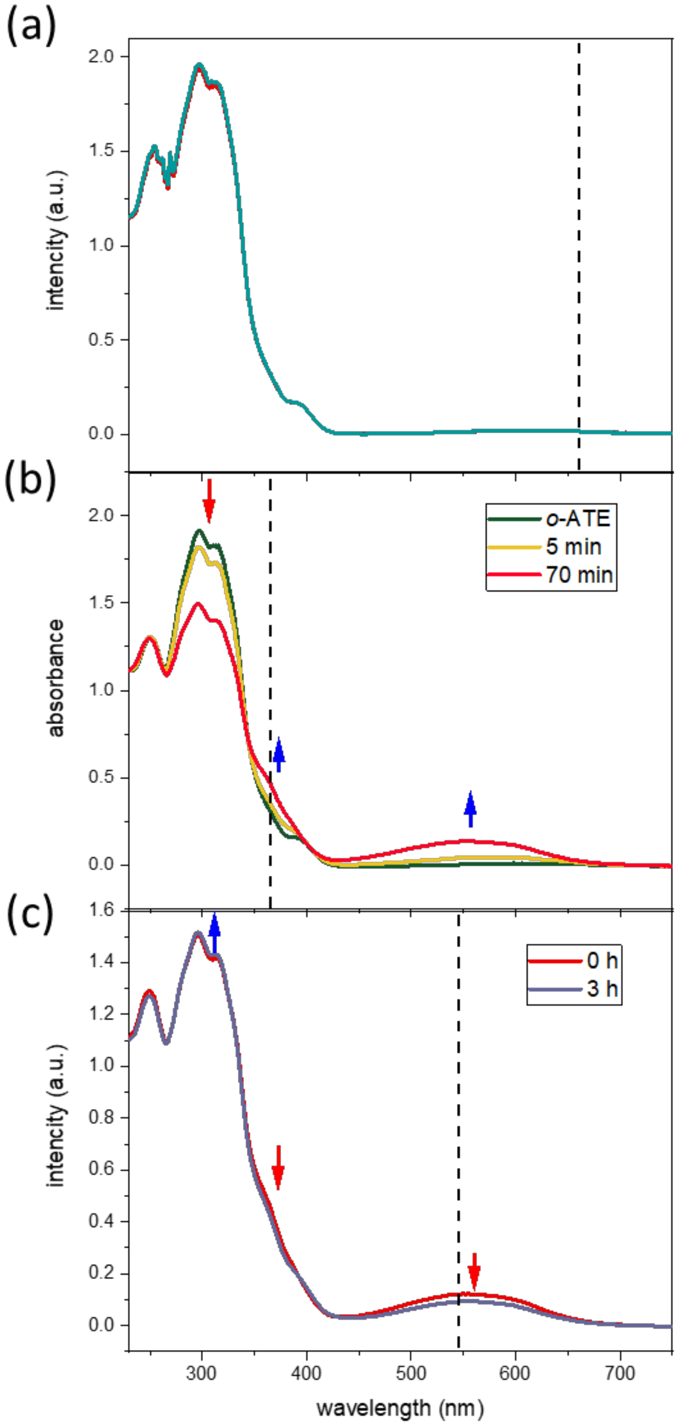

Figure S1. UV-vis absorption spectra of ***o*-ATE** (5.0×10^{-5} M in cyclohexane) after irradiation at (a) 660 nm for 16 h (red line) or (b) 365 nm for 70 min and (c) subsequent irradiation at 546 nm for 3 h. Irradiation wavelengths are indicated by dash lines.

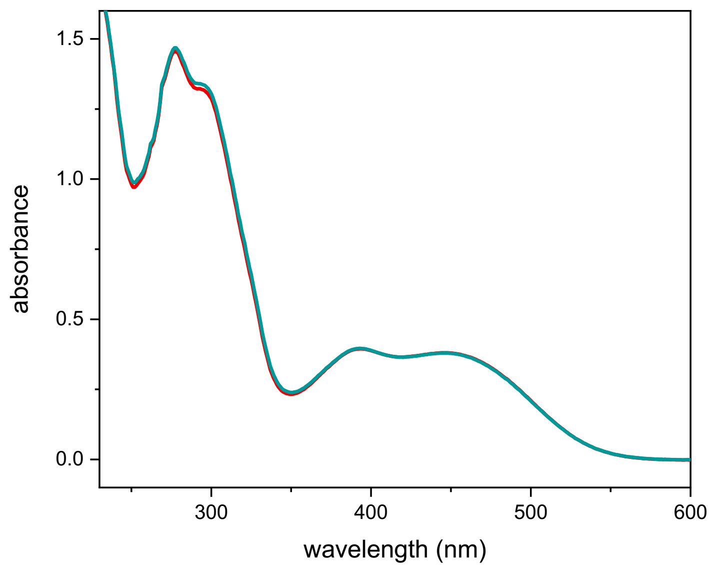

Figure S2. UV-vis absorption spectra of ***o*****-ATE-H**$^+$ ($5.0 \cdot 10^{-5}$ M in cyclohexane and $3.0 \cdot 10^{-2}$ M trifluoroacetic acid (TFA)) kept in dark for 16 h (red line).

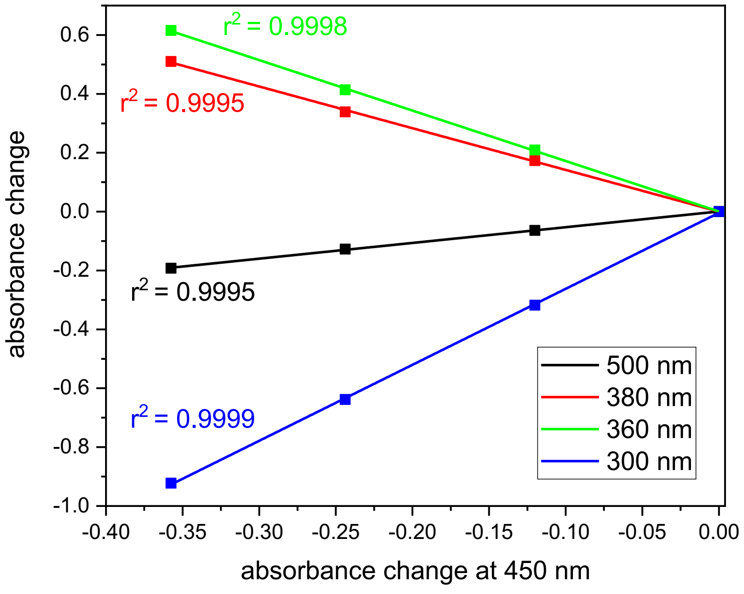

Figure S3. Mauser-type diagram and linear fit of spectral changes from Figure 2.

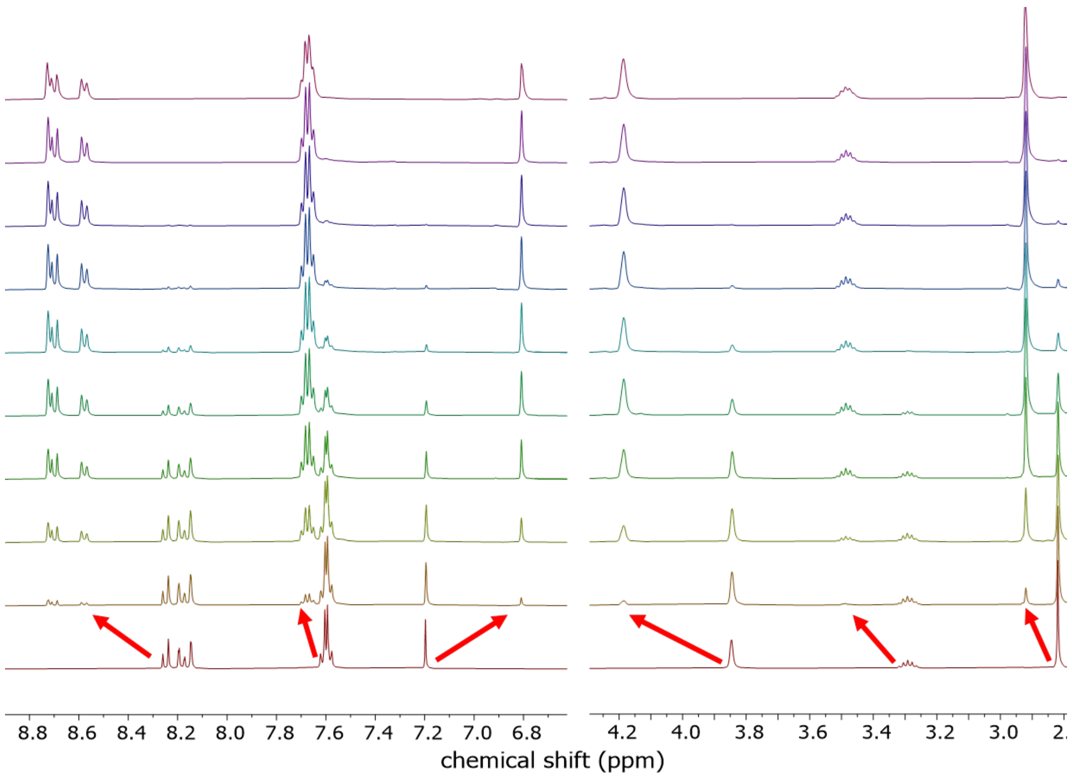

Figure S4. ^{1}H NMR spectra of ***o*-ATE-H⁺** at 248 K with the presence of 16 equiv. TFA under *in-situ* irradiation at 565 nm for 7 h to form ***c*-ATE-H⁺**. The interval between spectra are 45 min.

	o-ATE	c-ATE
**1**	0	48
**2**	108	79
**3**	146	26
**4**	96	45
**5**	123	18
**6**	94	55
**7**	127	31
**8**	104	57
**9**	29	-
**10**	99	73
**11**	125	22
**12**	110	84
**13**	35	16
**14**	86	-
**15**	197	133
**16**	99	0
**17**	116	115
**18**	255	not stable

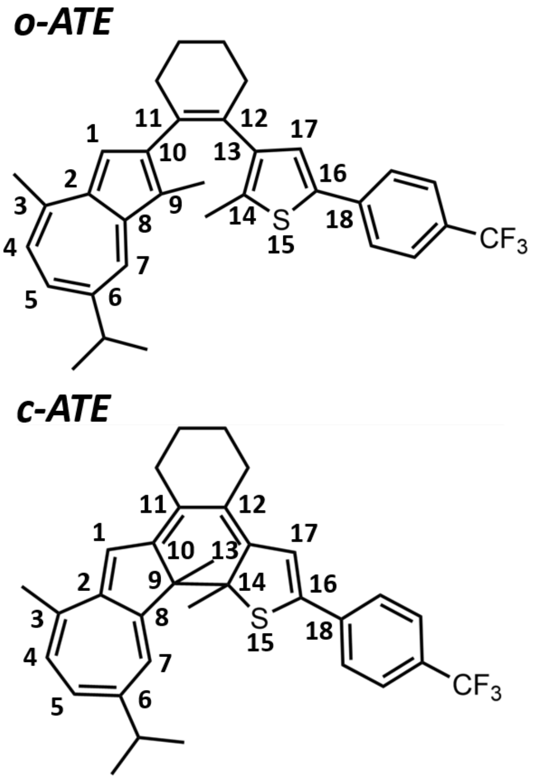

Figure S5. Relative electronic energies [kJ mol^{-1}] of **o-ATE** and **c-ATE** protonated at different positions based on B3LYP+D3//def2-TZVPP structure optimizations. Optimization of isomer 18 of c-ATE leads to the corresponding isomer 16.

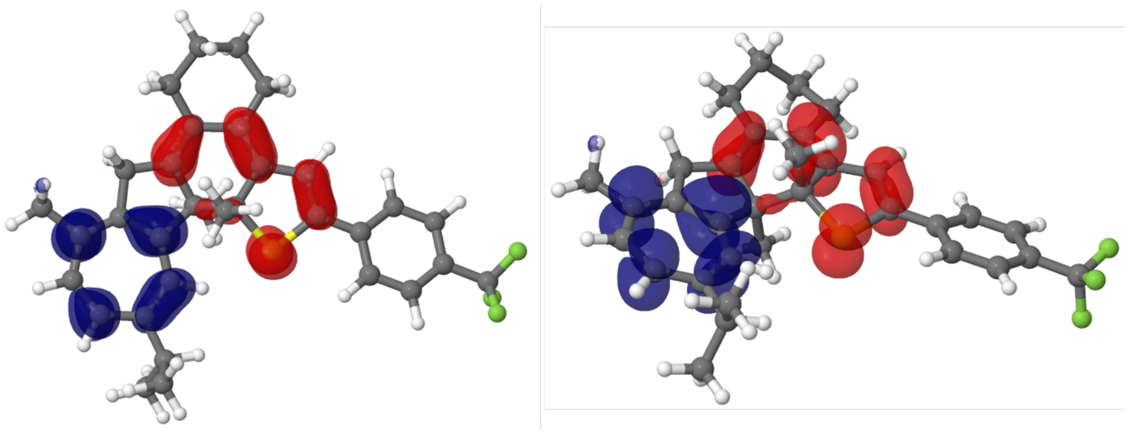

Figure S6. Calculated electron density shift (electron difference density) of the $S_0 \rightarrow S_1$ transition of ***c*-ATE-H$^+$** shown from two slightly different perspectives. Red and blue lobes refers to a decrease and increase, respectively, of electron density during excitation.

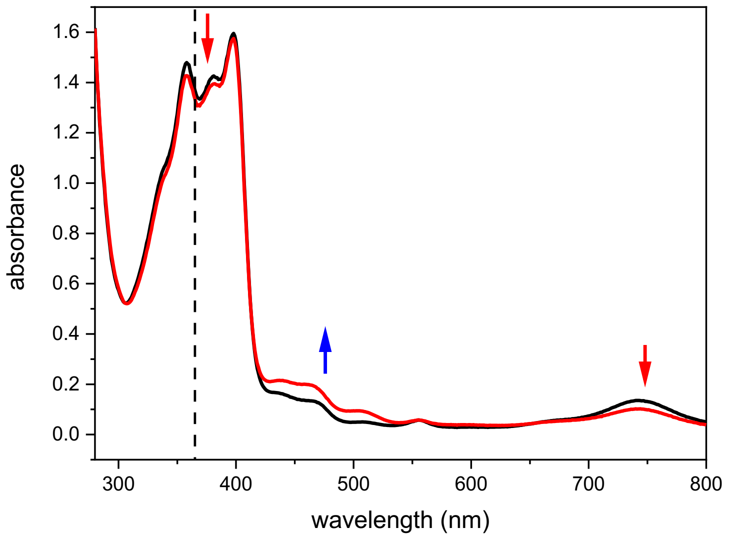

Figure S7. UV-vis absorption spectral change of ***c*-ATE-H**$^+$ ($5.0 \cdot 10^{-5}$ M in cyclohexane and $3.0 \cdot 10^{-2}$ M TFA) at −30 °C after irradiation at 365 nm for 85 min. Irradiation wavelength is indicated by a dashed line.

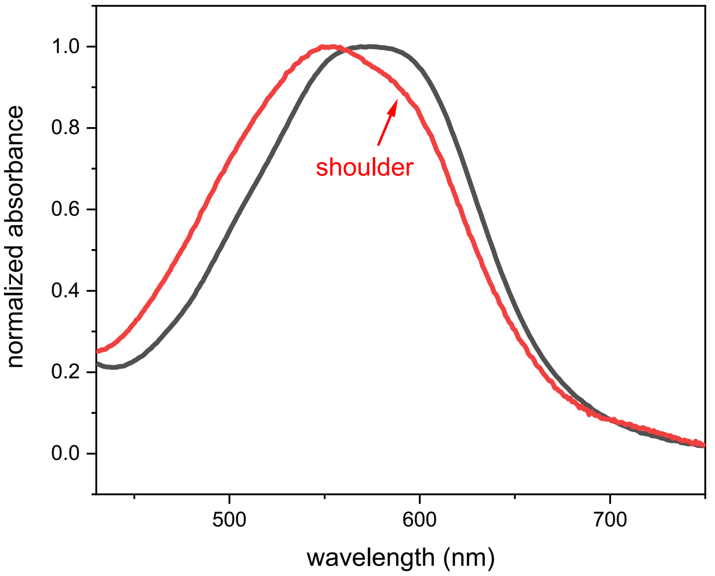

Figure S8. Normalized long wavelength UV-vis absorption spectra of ***c*-ATE** obtained from direct photoreaction (red line) or sequential protonation/photoreaction/neutralization (black line) of ***o*-ATE**. Note that in the former case the mixture was not in the photostationary state and contained large amount of ***o*-ATE**.

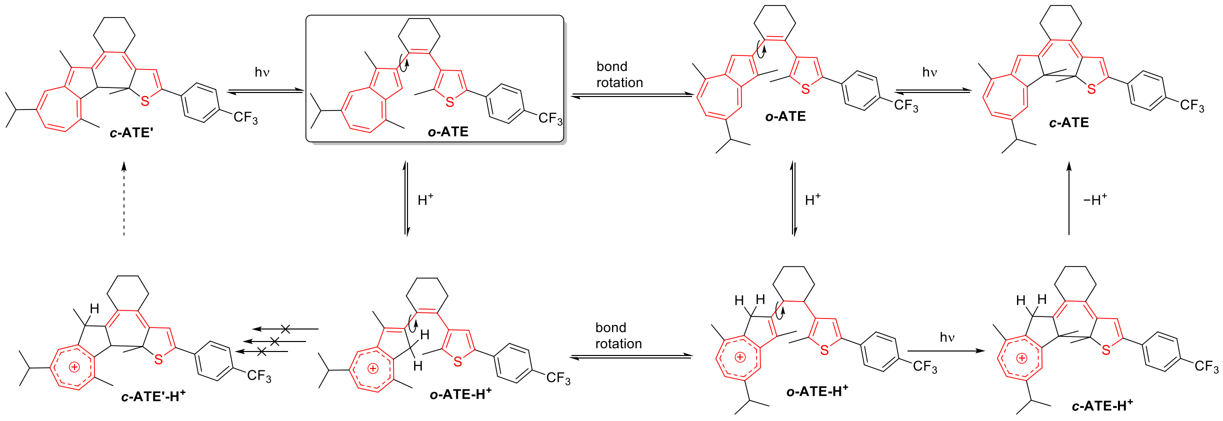

Figure S9. Isomer mixture and single isomer formed from direct photoreaction and sequential protonation/photoreaction/neutralization, respectively, of ***o*-ATE.**

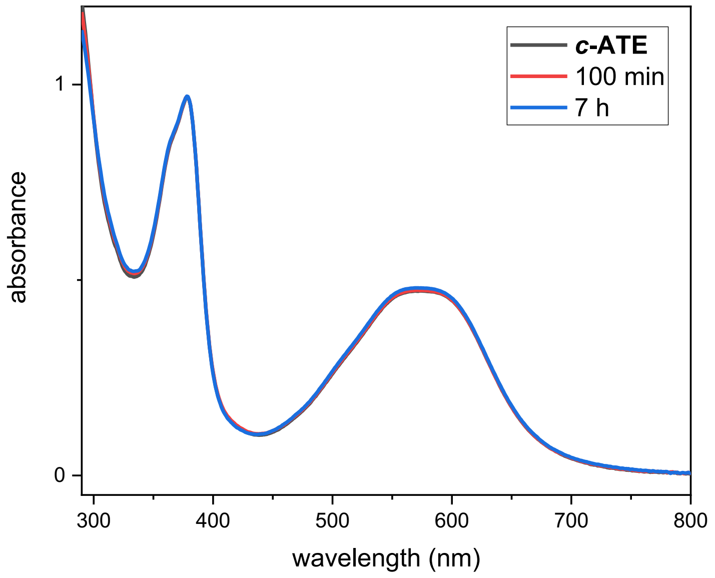

Figure S10. UV-vis absorption spectra of ***c*-ATE** kept in dark at room temperature for 7 h. the initial ***c*-ATE** was obtained from photoreaction of ***c*-ATE-H**$^+$ ($5.0 \cdot 10^{-5}$ M in cyclohexane and $3.0 \cdot 10^{-2}$ M TFA) and subsequent neutralization ($9.0 \cdot 10^{-2}$ M triethylamine (TEA)).

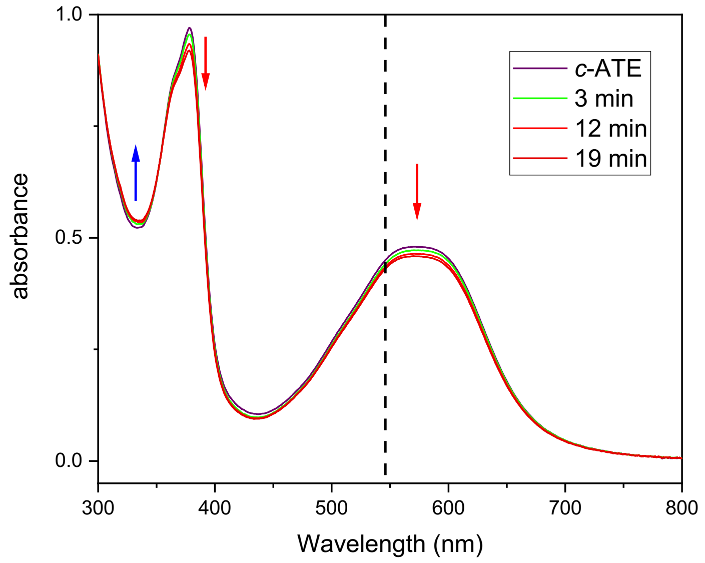

Figure S11. UV-vis absorption spectra of ***c*-ATE** upon irradiation at 546 nm for 19 min. The initial solution of ***c*-ATE** was obtained from photoreaction of ***c*-ATE-H$^+$** ($5.0 \cdot 10^{-5}$ M in cyclohexane and $3.0 \cdot 10^{-2}$ M TFA) and subsequent neutralization ($9.0 \cdot 10^{-2}$ M TEA). Irradiation wavelength is indicated by a dashed line.

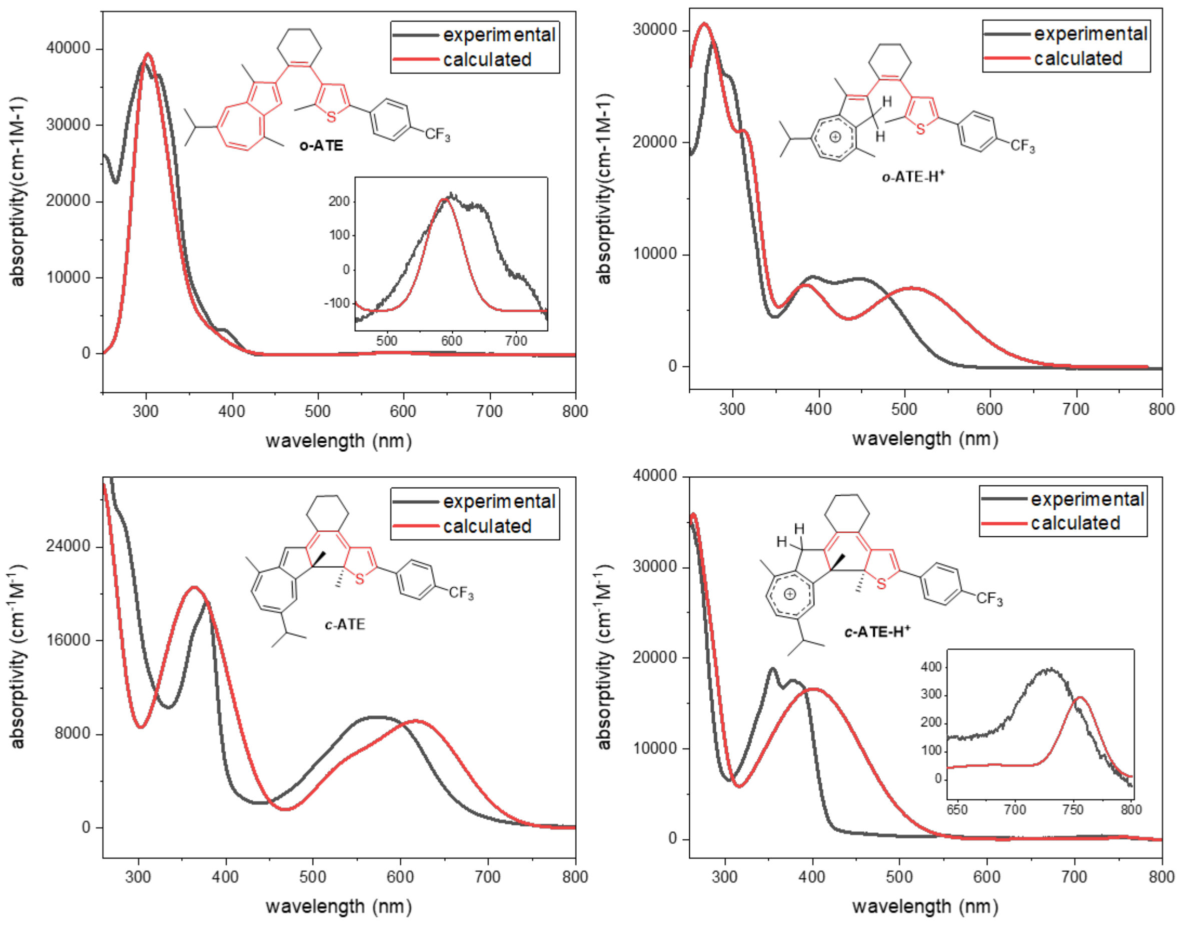

Figure S12. Experimental and calculated UV-vis absorption spectra of ***o*-ATE**, ***o*-ATE-H$^+$**, ***c*-ATE**, and ***c*-ATE-H$^+$**. Peak widths were obtained by Gaussian fits of calculated excitation energies and oscillator strengths on experimental spectra without any further constraints, yielding typical FWHM values of 30 – 130 nm.

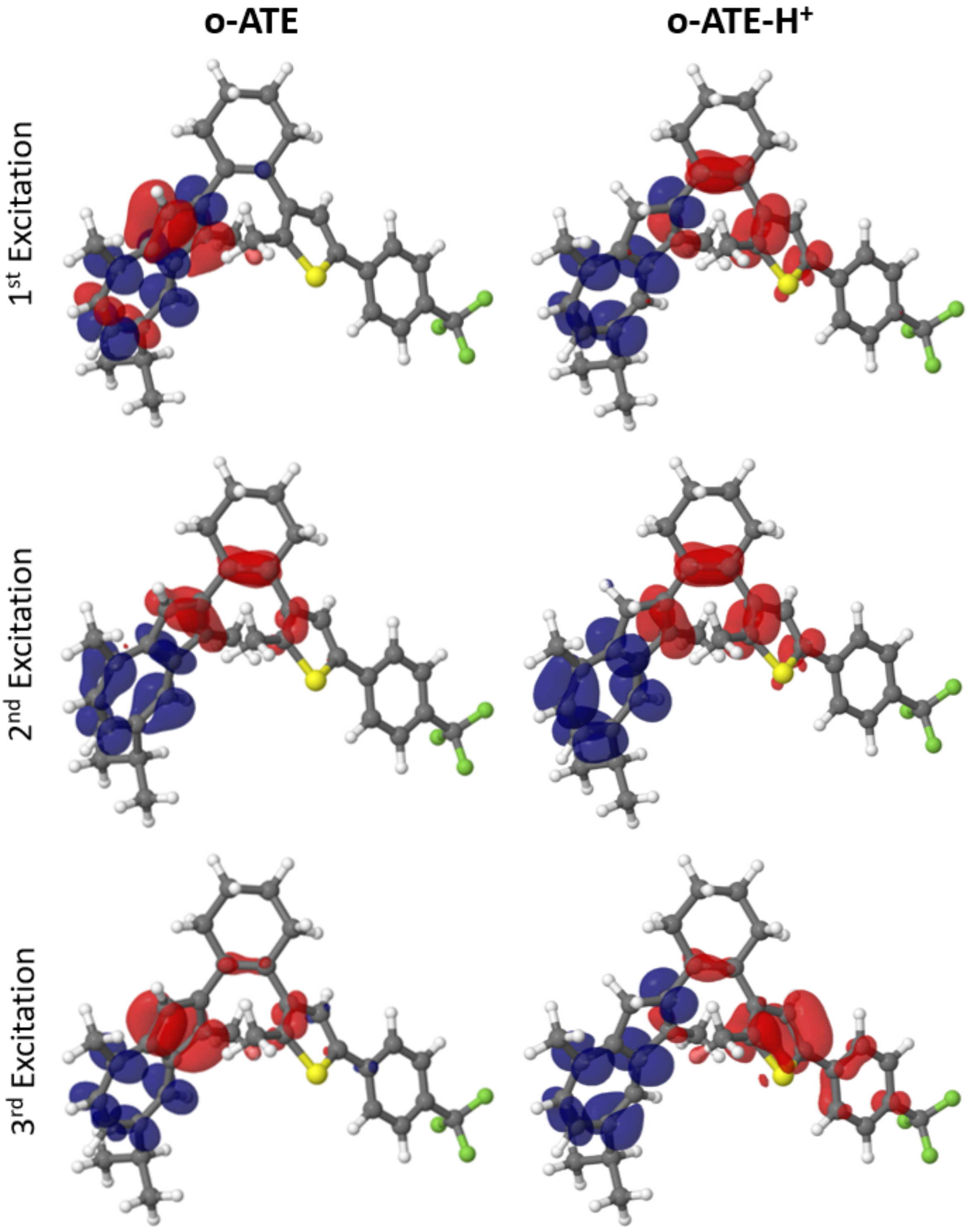

Figure S13. Electron difference densities of **o-ATE** and **o-ATE-H**$^{+}$ between ground state and first, second, and third excited states, respectively. Red lobes corresponds to a decrease of electron density and blue lobes to an increase of electron density during excitation.

NMR Spectra of New Compounds

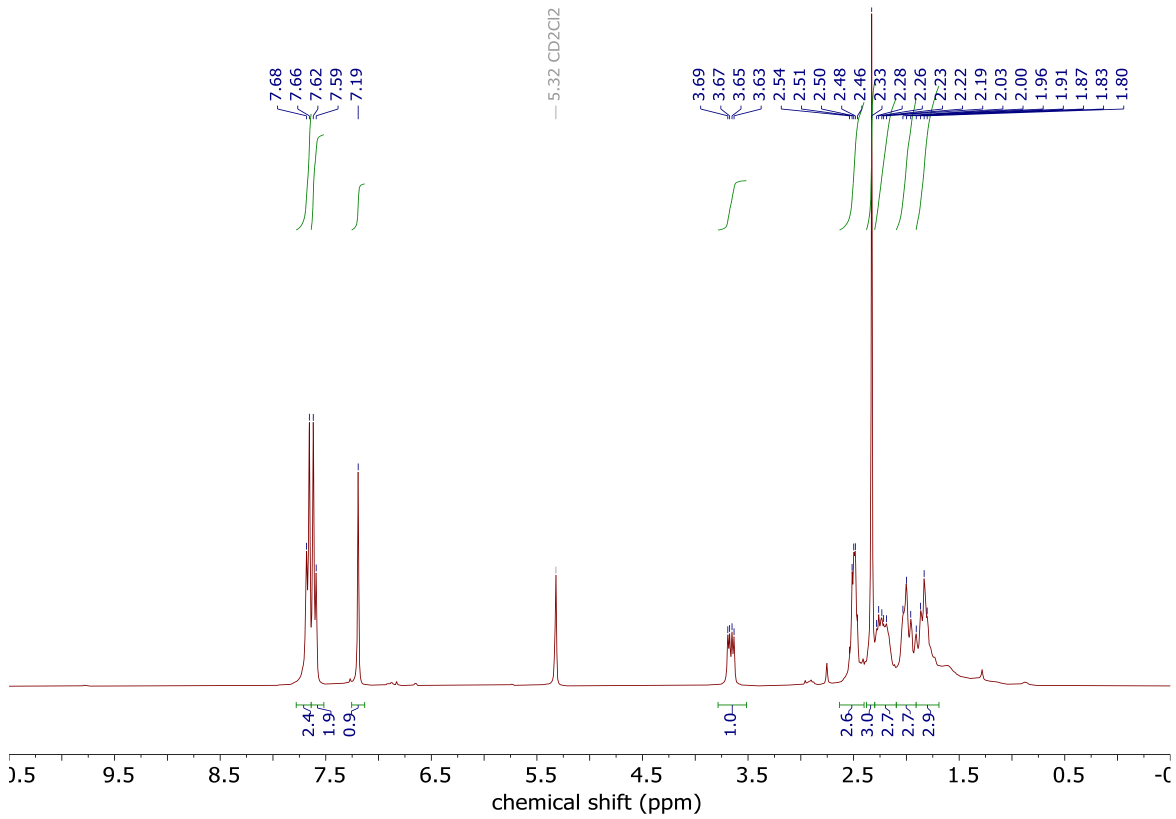

Figure S14. ^{1}H NMR spectrum of **2** (300 MHz, CD_2Cl_2).

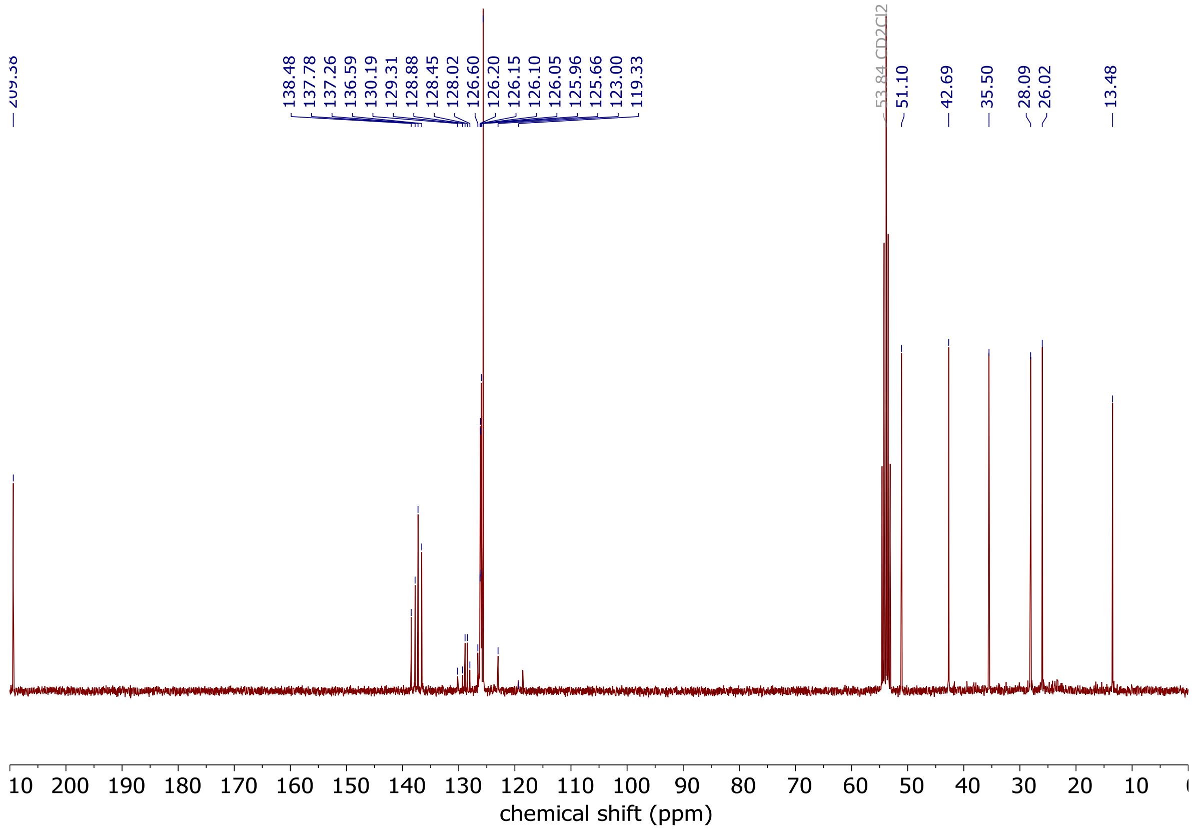

Figure S15. ^{13}C NMR spectrum of **2** (75 MHz, CD_2Cl_2).

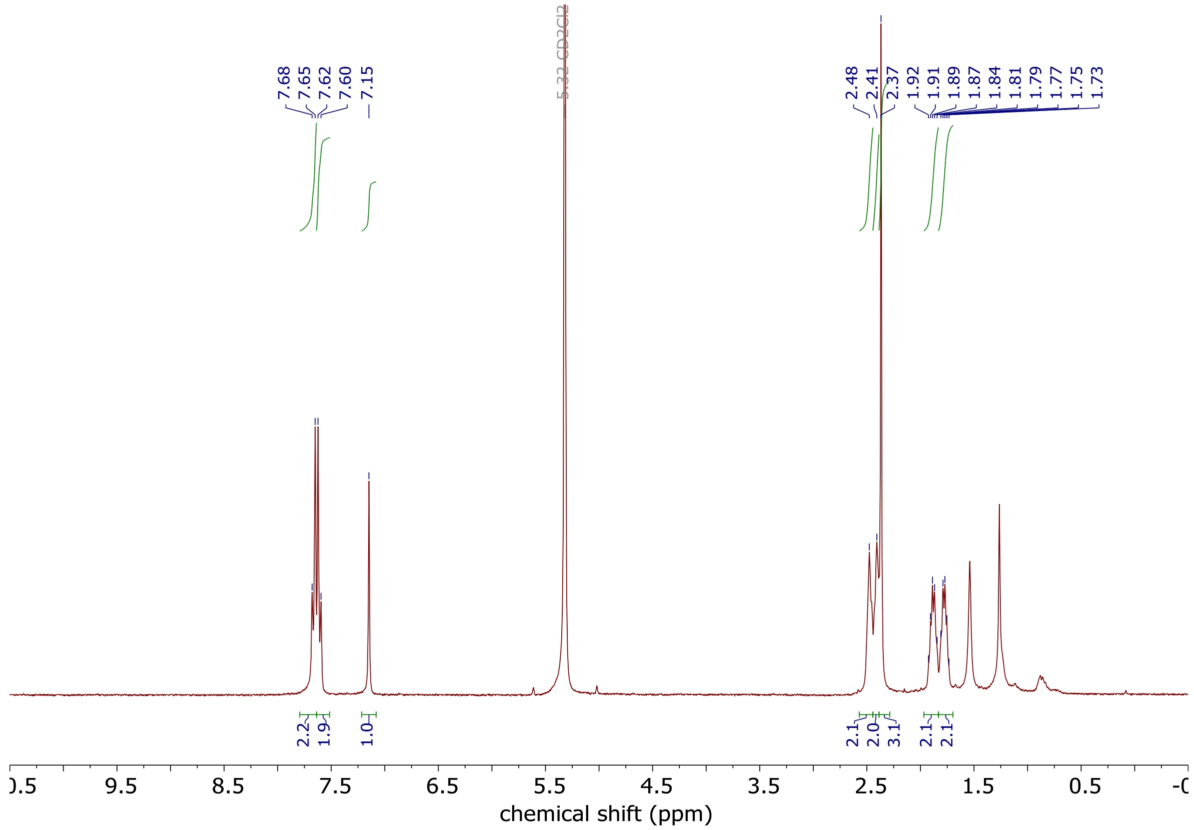

Figure S16. ^{1}H NMR spectrum of **3** (300 MHz, CD_2Cl_2).

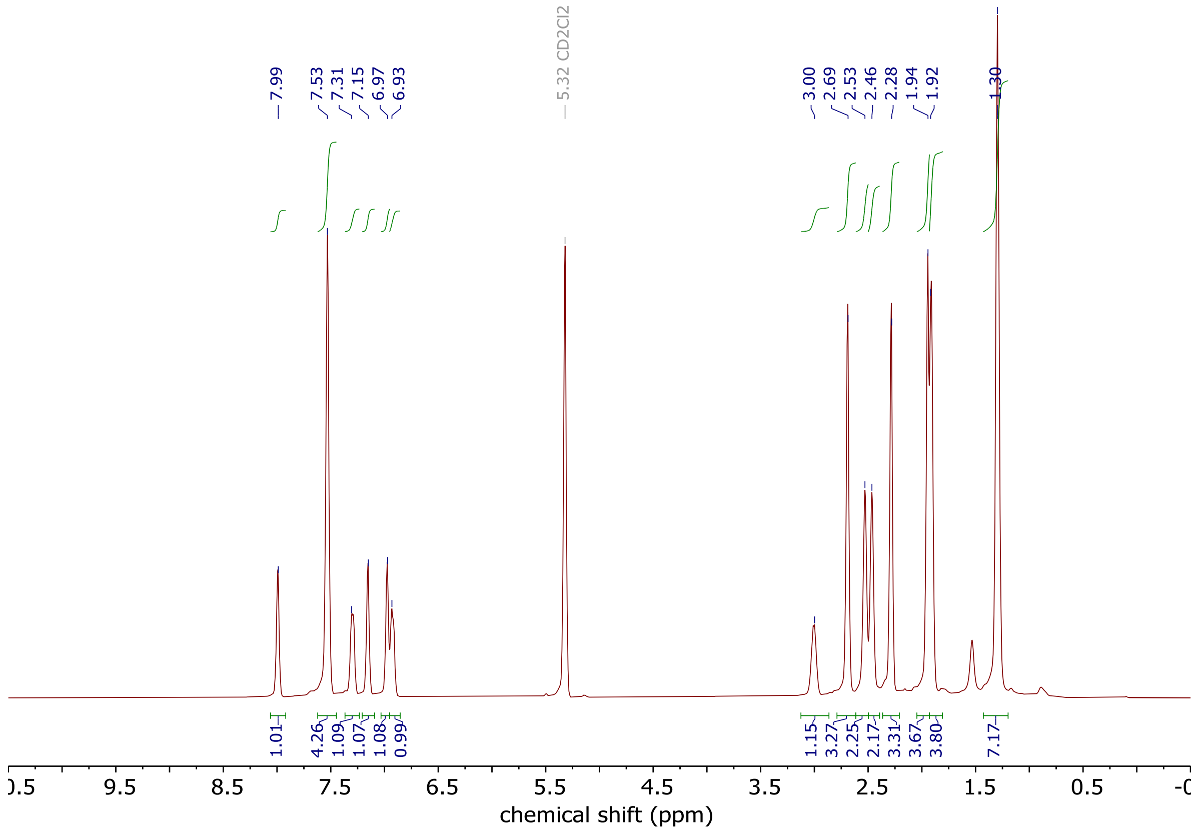

Figure S17. ^{1}H NMR spectrum of ***o*-ATE** (500 MHz, CD_2Cl_2).

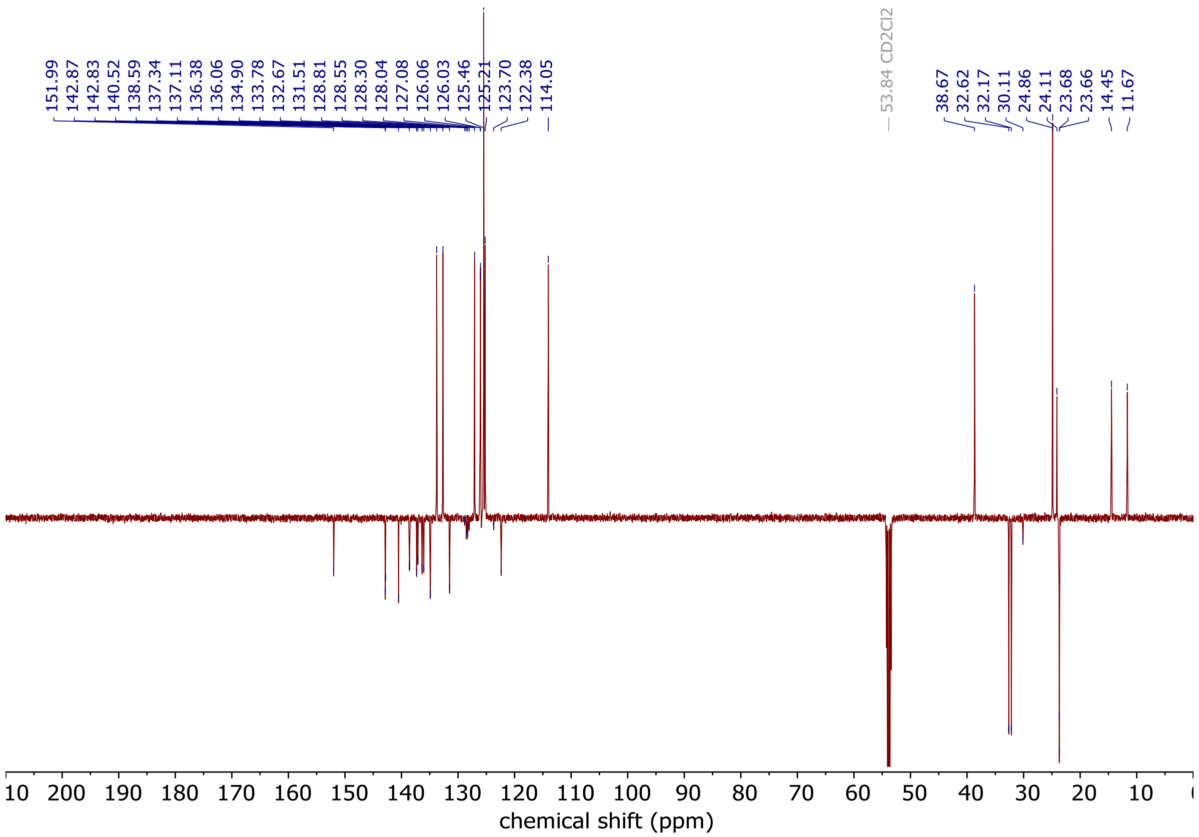

Figure S18. ^{13}C NMR APT spectrum of ***o*-ATE** (126 MHz, CD_2Cl_2).

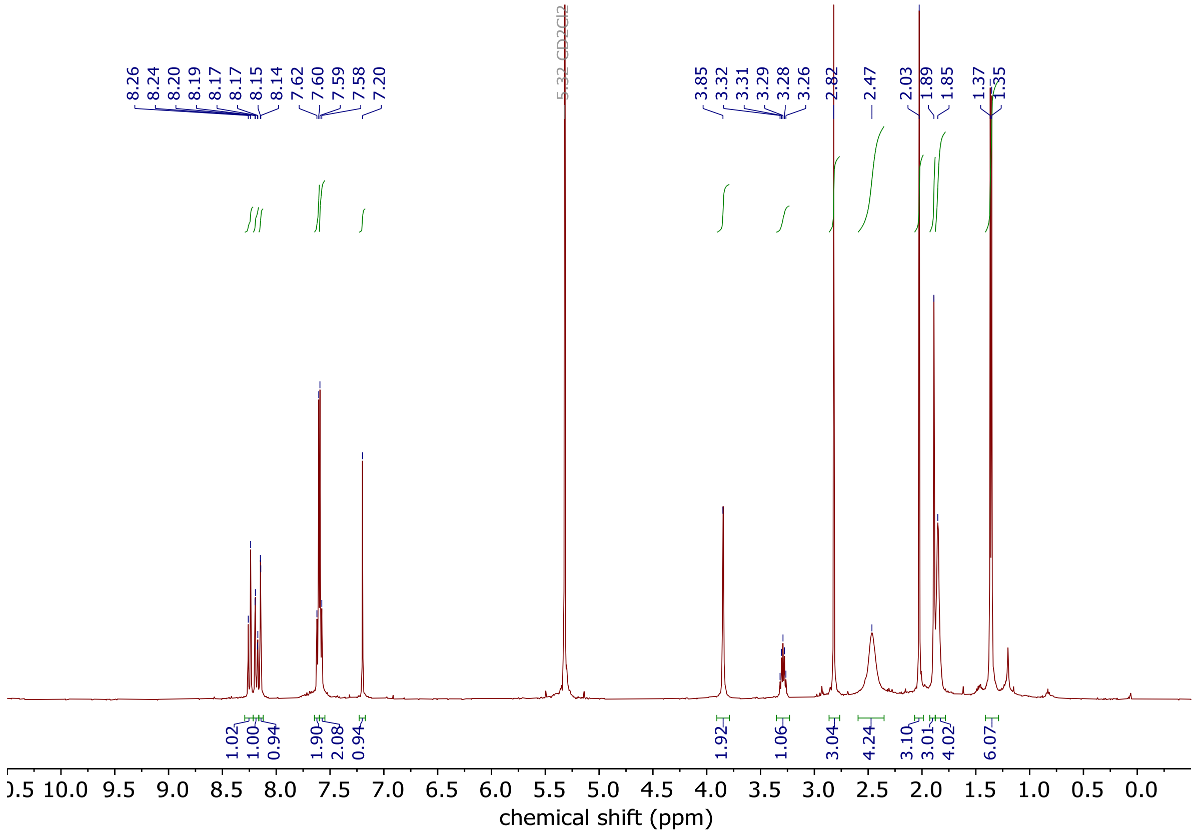

Figure S19. ^{1}H NMR spectrum of ***o*-ATE-H⁺** (500 MHz, CD_2Cl_2 + 16 equiv. TFA, 248 K).

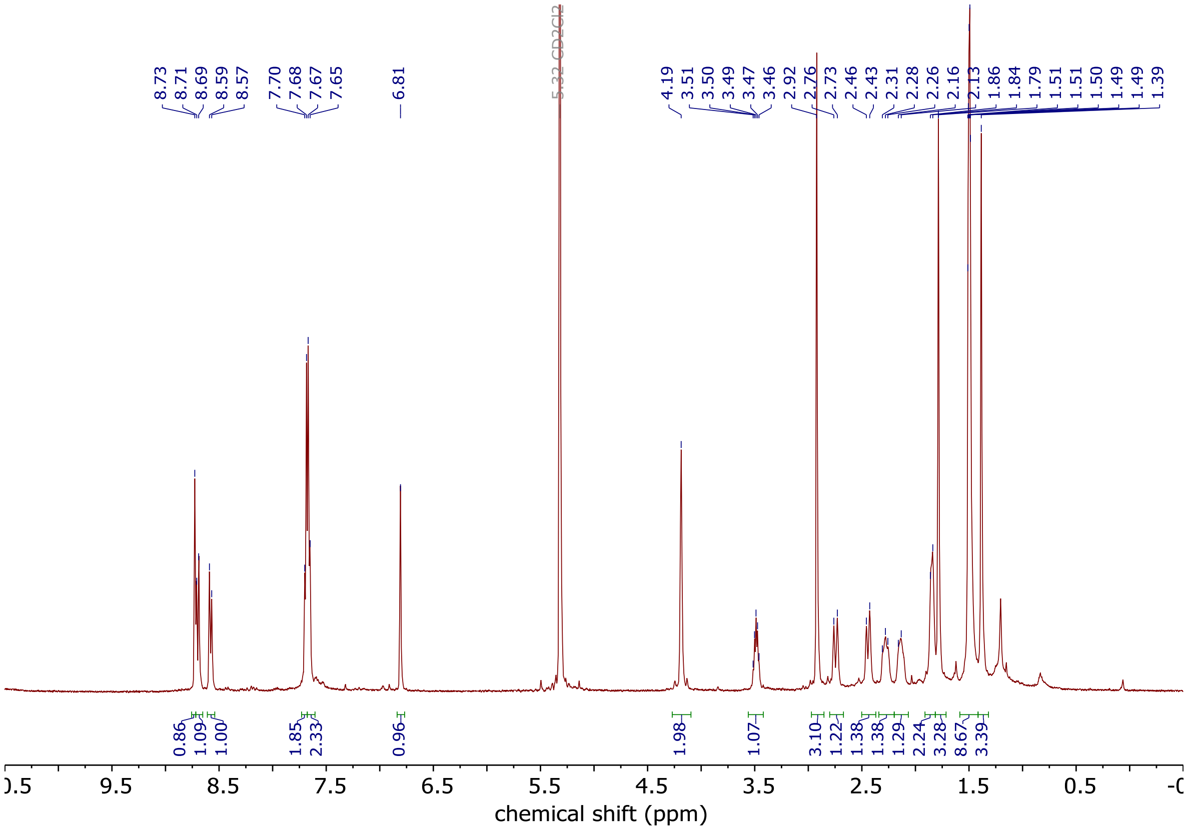

Figure S20. ^{1}H NMR spectrum of ***c*-ATE-H⁺** (500 MHz, CD_2Cl_2 + 16 equiv. TFA, 248 K).

References

[1] S. Fredrich, A. Bonasera, V. Valderrey, S. Hecht, *J. Am. Chem. Soc.* **2018**, *140*, 6432–6440.

[2] M. Narita, T. Murafuji, S. Yamashita, M. Fujinaga, K. Hiyama, Y. Oka, F. Tani, S. Kamijo, K. Ishiguro, *J. Org. Chem.* **2018**, *83*, 1298–1303.

[3] C. Jurissek, F. Berger, F. Eisenreich, M. Kathan, S. Hecht, *Angew. Chem. Int. Ed.* **2018**, DOI 10.1002/anie.201812284.

[4] F. Neese, *WIREs Comput. Mol. Sci.* **2012**, *2*, 73–78.

[5] F. Neese, *WIREs Comput. Mol. Sci.* **2018**, *8*, e1327.

[6] A. D. Becke, *J. Chem. Phys.* **1993**, *98*, 5648–5652.

[7] C. Lee, W. Yang, R. G. Parr, *Phys. Rev. B* **1988**, *37*, 785–789.

[8] S. Grimme, *J. Comput. Chem.* **2006**, *27*, 1787–1799.

[9] F. Weigend, R. Ahlrichs, *Phys. Chem. Chem. Phys.* **2005**, *7*, 3297.

[10] *Turbomole V7.0 2015*, University Of Karlsruhe And Forschungszentrum Karlsruhe GmbH, 1989 2007, TURBOMOLE GmbH, Since 2007. Available On Http://Www.Turbomole.Com, **n.d.**

[11] A. Schäfer, H. Horn, R. Ahlrichs, *J. Chem. Phys.* **1992**, *97*, 2571–2577.

[12] C. Jurissek, F. Berger, F. Eisenreich, M. Kathan, S. Hecht, *Angew. Chem. Int. Ed.* **2019**, *58*, 1945–1949.

[13] "Jmol: an open-source Java viewer for chemical structures in 3D. http://www.jmol.org/," **n.d.**
